# Electrodeposition of Al–Mg alloys from chloride-based molten salts

**DOI:** 10.1038/s41598-025-04094-1

**Published:** 2025-05-30

**Authors:** Sreesvarna Bhaskaramohan, Manepalli J. N. V. Prasad, G. V. Dattu Jonnalagadda, Sankara Sarma V. Tatiparti

**Affiliations:** 1 Department of Energy Science and Engineering, Indian Institute of Technology Bombay, Mumbai, 400076 India; 2 Department of Metallurgical Engineering and Materials Science, Indian Institute of Technology Bombay, Mumbai, 400076 India; 3 John F Welch Technology Centre, General Electric India Industrial Pvt. Ltd., Bangalore, 560066 India; 4 Materials Science and Engineering Department, University of Florida, Gainesville, FL 32611 USA

**Keywords:** Electrochemistry, Metals and alloys

## Abstract

Aluminum-magnesium (Al–Mg) alloys were potentiostatically electrodeposited on copper using Al anode at 180 °C from 62AlCl_3_ + 17NaCl + 15KCl + 6MgCl_2_ electrolyte. The depositions are categorized into two groups based on current density-time curves and deposit compositions. Group 1 corresponds to overpotentials from − 1.05 to − 1.10 V, and Group 2 from − 1.15 to − 1.30 V. Morphologies within Group 1 possess almost pure Al and coarsen with overpotential; while those in Group 2 possess Al and Mg, and refine with overpotential. Based on electrochemical, compositional, and spectral analyses, a generic deposition scheme is proposed. Mathematical variables in this scheme are adjusted rendering it suitable for both groups. Sources of Al for deposition are ${{\text{Al}}_{2}\text{Cl}}_{7}^{-}$, and ${\text{AlCl}}_{4}^{-}$; that of Mg is MgCl_2_ (possibly forming complexes in electrolyte). Al anodic dissolution occurs significantly in Group 1. Na(AlCl_4_) formation and Cl_2_ evolution occur at anode in Group 2. The proposed scheme is used to devise the following corollaries: (i) Al deposits preferentially in both groups; (ii) Mg deposition is prerequisite for Na(AlCl_4_) formation; (iii) Deposited Mg is curtailed to maximum of ~ 4.02 at.% by Na(AlCl_4_); and (iv) A minimum threshold exists for Al/Mg deposit composition ratios. The scheme and corollaries answer questions of practical and fundamental importance.

**Supplementary Information:**

The online version contains supplementary material available at 10.1038/s41598-025-04094-1.

## Introduction

Aluminum-magnesium (Al–Mg) alloys are important in several applications due to their lightweight, suitable mechanical properties and high corrosion resistance^1–3^. These properties are mainly governed by the alloy composition. For example, Al–Mg alloys containing up to about 5 wt% Mg are beneficial in heat exchangers for aerospace applications^1^. Lehmkuhl et al. deposited Al–Mg alloys containing 25 wt% Mg offering superior contact corrosion resistance^4^. Attaining desired compositions uniformly from conventional techniques such as rolling, extrusion, sub-rapid solidification, and casting is practically difficult^5–7^.

Electrodeposition offers flexibility in attaining desired compositions, morphologies, and crystallographic phases in deposits. However, electrodeposition of Al–Mg alloys is challenging as they cannot be deposited from aqueous solutions because standard reduction potentials of Al and Mg (${{E}^{0}}_{\text{Al}|{\text{Al}}^{3+}}$= –1.676 V and ${{E}^{0}}_{\text{Mg}|{\text{Mg}}^{2+}}$= –2.356 V vs NHE) are below water-splitting potential (${{E}^{0}}_{{\text{H}}_{2}\text{O}|{\text{OH}}^{-}}$= –0.828 V vs NHE)^8^. Hence, electrodeposition of Al–Mg was attempted from ionic liquids^9–13^, molten salts^14^, Grignard reagents^15–17^, and organometallics^4^. Up to 13.3 at.% Mg was deposited using AlCl_3_-MgBr_2_-butyraldehyde-benzene-LiAlH_4_^13^. Mayer’s work with electrolytes containing NaF, KF, Al(C_2_H_5_)_3_, Al(C_2_H_5_)_3_, tri-iso-butyl-aluminum (iBu_3_Al, Bu = C_4_H_9_) and Mg(C_2_H_5_)_2_ prepared from Grignard reagents yielded Al–Mg alloys at aluminum-alkyl/magnesium-alkyl mole ratio ≥ 3.5^16,17^. In our earlier work using Na[Al(C_2_H_5_)_4_] + 2Na[(C_2_H_5_)_3_Al-H-Al(C_2_H_5_)_3_] + 2.5Al(C_2_H_5_)_3_ + 6toluene, deposits with wide range of compositions and morphologies were obtained^18–20^. Crystallographically consistent facets, feather-like morphologies possess 1–7 at.% Mg^19^, smooth globules contain ~ 20 at.% Mg and rough globules forming over smooth globules possess ~ 65–80 at.% Mg^19^.

Molten salts are important for Al–Mg deposition as they are chemically simple and easily available, unlike organometallics, which are banned in some countries. However, they are less explored for Al–Mg deposition. In a standalone study, Li et al. obtained 2.41–9.14 at.% Mg using AlCl_3_-MgCl_2_-NaCl-KCl system^14^. A clear trend in deposit compositions with current densities/overpotentials; deposit-electrolyte composition relations; and Al–Mg deposition schemes from molten salts are yet to be established. The literature on pure-Al deposition claims that its sources for deposition are ${{\text{Al}}_{2}\text{Cl}}_{7}^{-}$ or/and ${\text{AlCl}}_{4}^{-}$ in molten salts^21–25^. Such information can help in investigating the scheme of Al–Mg deposition from molten salts.

Here, Al–Mg alloys were electrodeposited potentiostatically at different overpotentials and 180 °C on Cu cathode by employing Al anode and chloride-based molten salt electrolytes. Based on the observed current density (*i*)-time (*t*) trends, the depositions can be segregated into two groups. Through the analysis of the deposit compositions and spent electrolytes, cyclic voltammetry, anodic dissolution, and salt formations at anode, an Al–Mg deposition scheme is proposed and important corollaries are devised. These scheme and corollaries are used to answer the following questions: (i) Can a desired content of Mg be obtained in the deposits? Alternatively, can Al–Mg alloys be deposited with any composition? and (ii) how can the deposit composition be controlled?

## Results and discussion

### Cyclic voltammetry

**Fig. 1:**
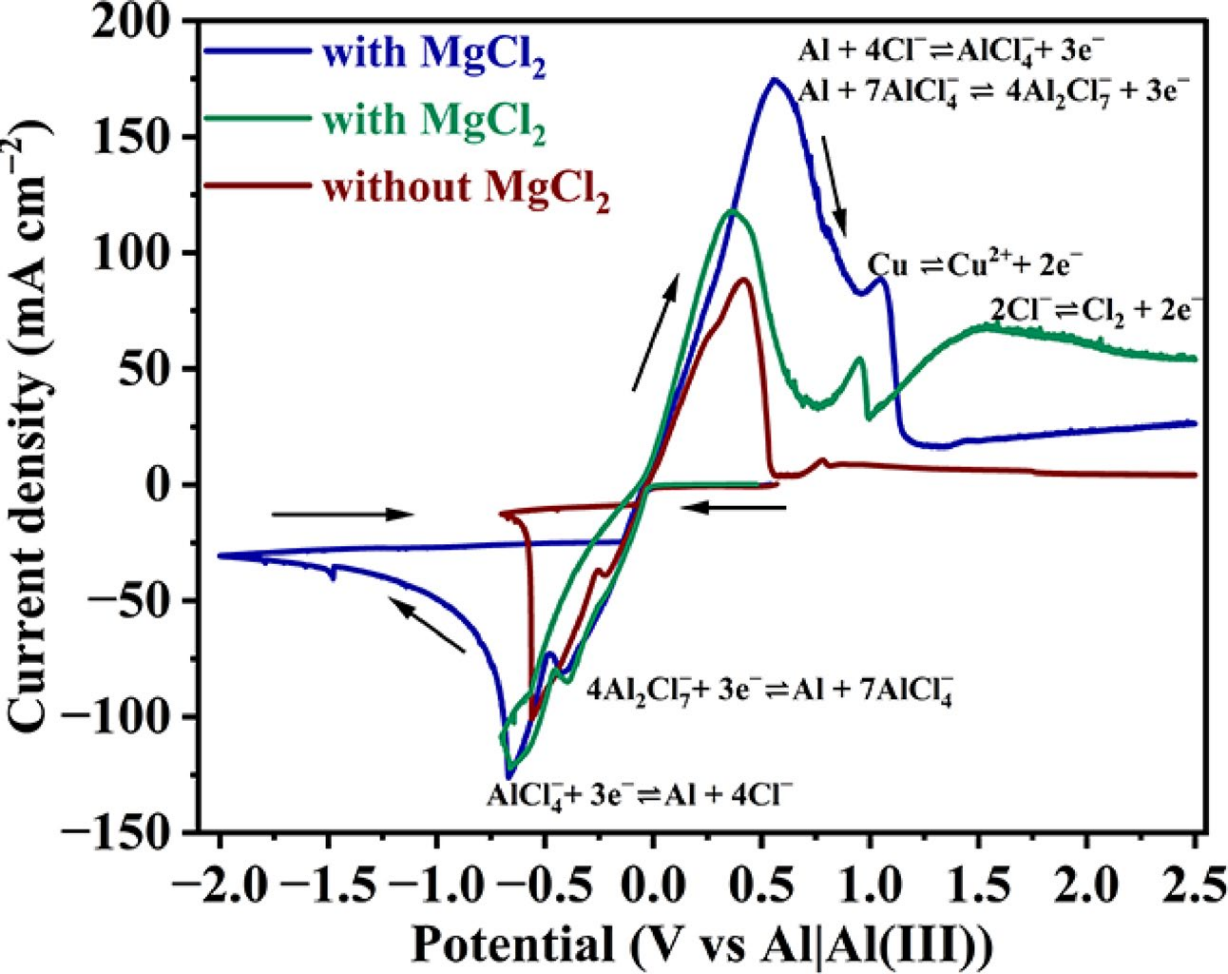
Cyclic Voltammograms on Cu using electrolytes with (62AlCl_3_ + 17NaCl + 15KCl + 6MgCl_2_) and without (61AlCl_3_ + 22NaCl + 17KCl) magnesium chloride.

The potential window for electrodeposition and the possible peaks due to various electrochemical reactions for deposition were checked by cyclic voltammetry (CV) in electrolytes with and without MgCl_2_ (Fig. 1). The CVs show two overlapping cathodic peaks with peak potentials around − 0.22 and − 0.66 V vs Al|Al(III) that correspond to Al deposition from ${{\text{Al}}_{2}\text{Cl}}_{7}^{-}$ (${{4\text{Al}}_{2}\text{Cl}}_{7}^{-}+ {3\text{e}}^{-}\rightleftharpoons \text{Al}+{7\text{AlCl}}_{4}^{-}$)^22,26^, and ${\text{AlCl}}_{4}^{-}$ (${\text{AlCl}}_{4}^{-}+ {3\text{e}}^{-}\rightleftharpoons 3\text{Al}+{4\text{Cl}}^{-}$)^27^, respectively. The corresponding electrochemical reactions are given near these peaks in Fig. 1. From these cathodic peaks in Fig. 1, the reduction of ${{\text{Al}}_{2}\text{Cl}}_{7}^{-}$ deposits Al and produces ${\text{AlCl}}_{4}^{-}$. This ${\text{AlCl}}_{4}^{-}$, along with that already present in the electrolyte, reduces to deposit further Al. Hence, the peak current density of ${\text{AlCl}}_{4}^{-}$ reduction can be higher than that of the reduction of ${{\text{Al}}_{2}\text{Cl}}_{7}^{-}$. The theoretical reversible potential for Mg deposition is − 0.68 V vs Al|Al(III)^8^. Hence, the deposition of Mg may not be significant in CV from the electrolyte containing MgCl_2_ when the switching potential is just − 0.7 V vs Al|Al(III). Hence, a separate CV was conducted from the electrolyte containing MgCl_2_ by extending the switching potential to − 2.00 V vs Al|Al(III) to facilitate Mg deposition and to select potentials for potentiostatic Mg deposition. The current densities within this extended range are higher (~ − 25 mA cm^−2^) than those (~ − 10 mA cm^−2^) in the CV without MgCl_2_ (switching potential: − 0.7 V vs Al|Al(III)), indicating the deposition of more metal within this extended range of potentials. However, the peaks corresponding to Mg deposition are not discernible in the cathodic segment, possibly owing to its negligible contribution to deposition. For the present study the applied potentials for Al–Mg deposition were selected between − 0.55 and − 1.0 V vs Al|Al(III).

Two anodic peaks are seen in all the CVs. The first peak spans a broad range of potentials. The second peak appears at around + 0.95 or + 1.07 V vs Al|Al(III) in the CV from the electrolyte with MgCl_2_; and at around + 0.78 V vs Al|Al(III) in the CV from the electrolyte without MgCl_2_ (Fig. 1). This second peak in all the CVs is due to the Cu (working electrode (W.E.)) dissolution as given in Supplementary Fig. S1. Cu dissolution was confirmed by two separate Linear Sweep Voltammetry (LSV) experiments from open circuit potential (OCP) to + 2.50 V vs Al|Al(III) with Cu and Pt as the working electrodes, shown in Supplementary Fig. S1. The LSV curve with Cu W.E. showed an anodic peak at around + 1.38 V vs Al|Al(III); whereas, it is absent when Pt is W.E.—confirming that the anodic peak at around + 1.07 V vs Al|Al(III) in Fig. 1 is due to Cu dissolution.

The first anodic peak in all the CVs in Fig. 1 is broad and not symmetric suggesting convolution of multiple oxidation reactions corresponding to Al dissolution. Yao et al. also reported the convoluted anodic peak corresponding to Al dissolution^23^. The presence of two cathodic peaks (due to deposition of Al) in Fig. 1 justifies two anodic peaks due to Al dissolution within this convolution. Al dissolution through two peaks is shown by Stafford et al^27^. Most likely here, the first portion in the convoluted peak is due to formation of ${\text{Al}}{\text{Cl}}_{4}^{-}$ ($\text{Al}+{4\text{Cl}}^{-}\rightleftharpoons {\text{AlCl}}_{4}^{-}+ {3\text{e}}^{-}$)^27^ and the second peak is due to the formation of${\text{Al}}_{2}{\text{Cl}}_{7}^{-}$ ($\text{Al}+{7\text{AlCl}}_{4}^{-} \rightleftharpoons {{4\text{Al}}_{2}\text{Cl}}_{7}^{-}+ {3\text{e}}^{-}$)^22,26^. The highest anodic peak current density in the CV from MgCl_2_ containing electrolyte with extended switching potential (− 2.00 V vs Al|Al(III)) is because of the highest metal dissolution. Interestingly, even when the switching potential is − 0.7 V vs Al|Al(III), the anodic current densities are slightly higher in the CV from the electrolyte containing MgCl_2_ than those in the absence of it. This indicates that slightly more metal deposited in the cathodic segment in the CV from the electrolyte containing MgCl_2_ dissolved within the potentials under the anodic peaks.

The CV from the electrolyte containing MgCl_2_ develops undulations and rises in current density after the second peak (Fig. 1). Such a rise in the current density was attributed to Cl_2_ evolution according to Hong-Min et al.^21^. The oxidation reaction pertaining to Cl_2_ evolution (${4\text{AlCl}}_{4}^{-}\rightleftharpoons {{2\text{Al}}_{2}\text{Cl}}_{7}^{-}+ {\text{Cl}}_{2}\left(\uparrow \right)+2{e}^{-})$^21,24^ as mentioned by Hong-Min et al.^21^ is a combination of two reactions which will be discussed in the Scheme of deposition section of the present study. This signature is absent in the CV from the electrolyte without MgCl_2_ (Fig. 1).

### Current density-time curves

**Fig. 2:**
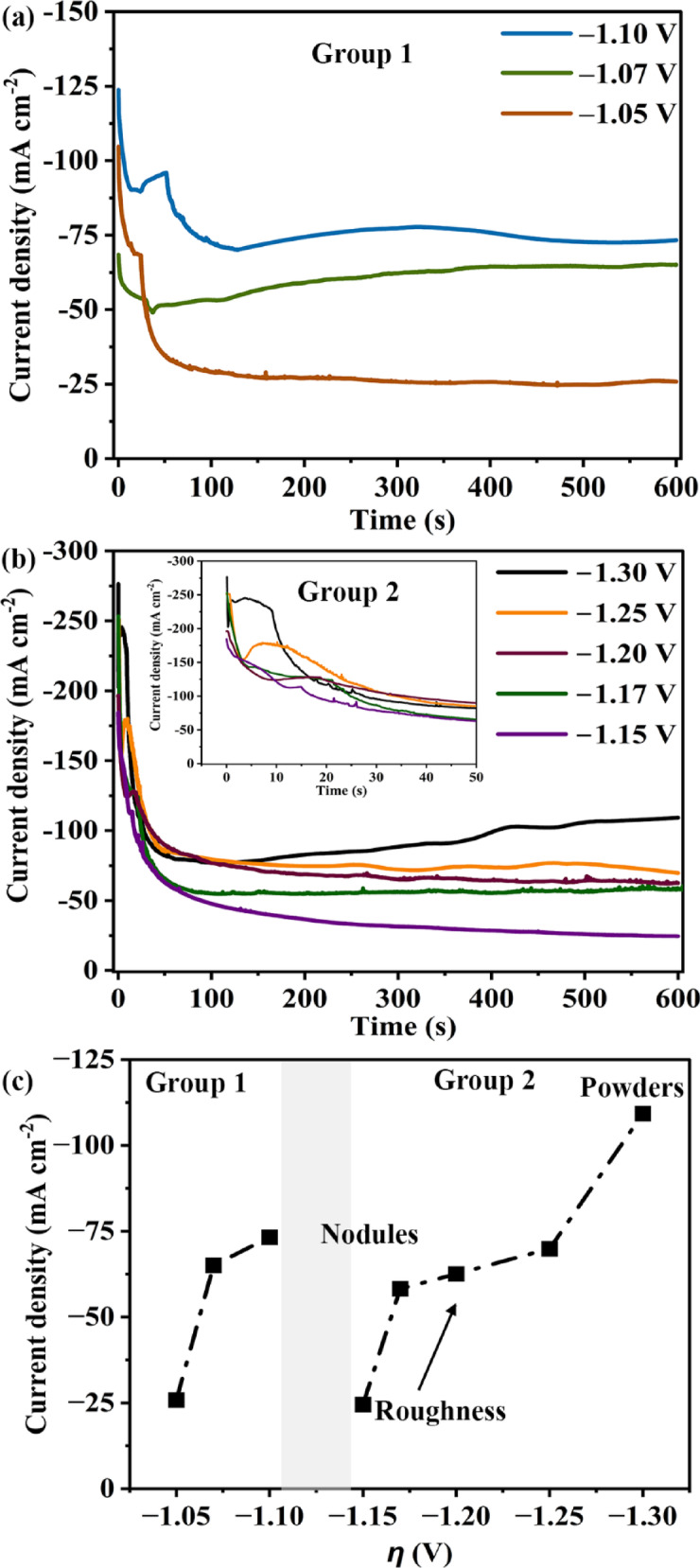
Current density-time curves of Al–Mg electrodeposition on Cu using 62AlCl_3_ + 17NaCl + 15KCl + 6MgCl_2_ at overpotentials between: (**a**) − 1.05 and − 1.10 V; (**b**) − 1.15 and − 1.30 V; and (**c**) steady-state current densities versus overpotential (*η*).

Figures 2a, b show the current density (*i*)*-*time (*t*) curves of all the Al–Mg depositions. All the *i*–*t* curves show typical phases of deposition. The first part of *i*–*t* curve is due to the charging current and decays as the nucleation starts. The following increase in current densities is due to the growth of nuclei. Eventually, the diffusion zones of the nuclei which are mutually adjacent overlap leading to linear mass transfer and planar surface. This is reflected by the decreasing current before attaining steady state^28^. Normally, the current densities are expected to be higher (in magnitude) at higher overpotentials^29^. Contrarily, here the steady-state current densities from − 1.15 to − 1.25 V are either lower or comparable to that at − 1.10 V. This necessitates the *i*–*t* curves to be categorized into two groups viz. Group 1 and 2. Group 1 consists of the *i*–*t* curves between − 1.05 and − 1.10 V; and, those between − 1.15 and − 1.30 V belong to Group 2. Within each of these groups, the steady-state current densities increase with an increase in the magnitude of overpotential, maintaining consistency. The results of the electrodepositions at overpotentials within − 1.10 and − 1.15 V inconsistently (i.e. without any trend) fall into either of these groups leading to ambiguity. For instance, the *i*–*t* curves of multiple attempts of deposits at an overpotential of − 1.12 V are shown in Supplementary Fig. S2. These curves are inconsistent and fail to show any trend. Hence, the overpotentials between − 1.10 and − 1.15 V are discarded from the present analysis.

The steady-state current densities at the end of the potentiostatic deposition experiments (i.e. 600 s) are plotted versus overpotential for all the depositions in Fig. 2c. From Fig. 2c, similar current densities between ~ 30 and ~ 75 mA cm^−2^ can be obtained using multiple potentials. Normally, a given overpotential renders a unique current density value, when the rest of the parameters are unchanged^29^. However, the lack of uniqueness here suggests a change in scheme from Group 1 to Group 2. Moreover, the plot pertaining to Group 2 in Fig. 2c suggests a plateau-like feature in current density (between ~ 60 and ~ 75 mA cm^−2^). Such a plateau-like feature is absent in Group 1, due to the switchover to Group 2 at further higher overpotentials.

### Morphologies of deposits

**Fig. 3:**
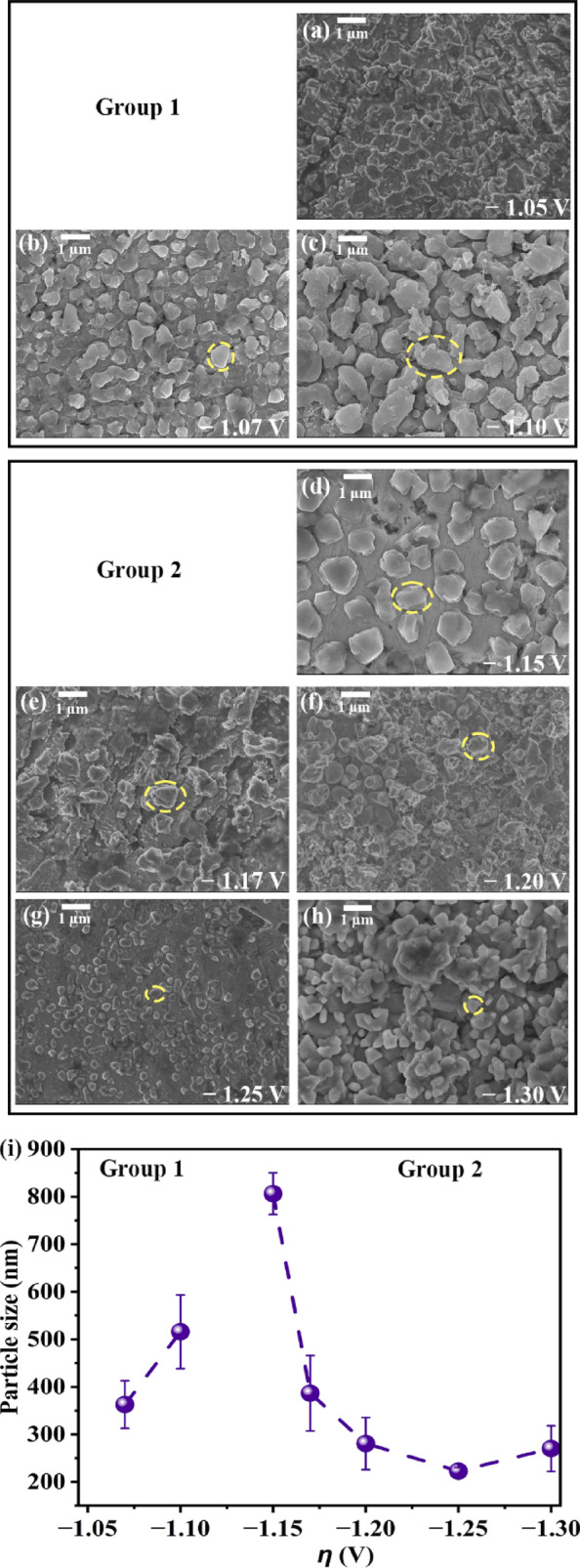
Morphologies of deposits at overpotentials within: (**a**–**c**) Group 1; and (**d**–**h**) Group 2; and (**i**) Particle size versus overpotential (*η*).

Figure 3 shows the morphologies of deposits. Within any given deposit the morphology is uniform, which is a characteristic of potentiostatic deposition^20^. Generally, morphologies are governed by overpotentials/current densities and deposit compositions^19,30,31^. Since all these morphologies are characterized after deposition, they represent the scenarios in the steady-state current density regimes (Fig. 2a–c). The morphology of the deposit at − 1.05 V covers W.E. At higher overpotentials the growth-features (referred to as “particles”, encircled in Fig. 3b–h) are prominent. The average particle sizes and their standard deviations (estimated for at least 200 particles from each of the deposits through multiple SEM images) are shown in Fig. 3i. From Fig. 3i the particles coarsen with overpotential within Group 1. This is because, at these potentials, the resultant current densities are much higher than that at − 1.05 V (Fig. 2a), where the deposition rates are faster. At faster rates, the depositing ions have insufficient time for surface diffusion. Hence, while depositing they accumulate over the existing deposition front and grow. Similar observations of Al–Mg deposits using molten salt were reported before^14^.

In Group 2, the particles refine from − 1.15 to − 1.30 V (Fig. 3d–h). A closer observation of the *i*–*t* curves in Group 2 reveals that the maximum current densities during nucleation are higher at larger overpotentials (inset, Fig. 2b). This trend is absent in Group 1. Such higher current densities lead to higher nucleation density^32^, leading to particle refinement from − 1.15 to − 1.30 V (Fig. 3i). Moreover, the steady-state current densities of depositions at − 1.17, − 1.20, and − 1.25 V are comparable and reasonably higher than at − 1.15 V (Fig. 2b, Fig. 2c). This corroborates with the relatively closer average sizes of their particles compared with that at − 1.15 V (Fig. 3i). The current densities usually manifest as the charge that gets transferred on to the substrate during deposition. The charge densities of the deposits were estimated (for 600 s of deposition duration) by integrating the *i*–*t* curves at all the potentials and are listed in Table 1. The nominal thicknesses were estimated by using the deposit weights and the compositions and are also shown in Table 1. The charge densities increase with overpotential within each of the groups due to the increase in the current density as expected. Accordingly, the deposit thickness also increases within these groups. The trend in charge density corroborates with that in steady-state current density as function of overpotential (Table 1 and Fig. 2c). The different trends in the particle sizes, charge and steady-state current densities with overpotentials across groups 1 and 2 suggests different nucleation-growth behaviors across these groups. A detailed study on the nucleation-growth behavior of Al–Mg alloys will be published elsewhere.

**Table 1:** Charge density and nominal thickness at different overpotentials.

Group	Overpotential (V)	Charge density (C cm^−2^)	Nominal thickness (μm)
1	− 1.05	15.4	5.3
	− 1.07	35.0	12.1
	− 1.10	42.7	14.7
2	− 1.15	21.7	7.5
	− 1.17	30.2	10.4
	− 1.20	42.9	14.9
	− 1.25	43.0	15.0
	− 1.30	52.1	18.4

### Compositional trends

**Fig. 4:**
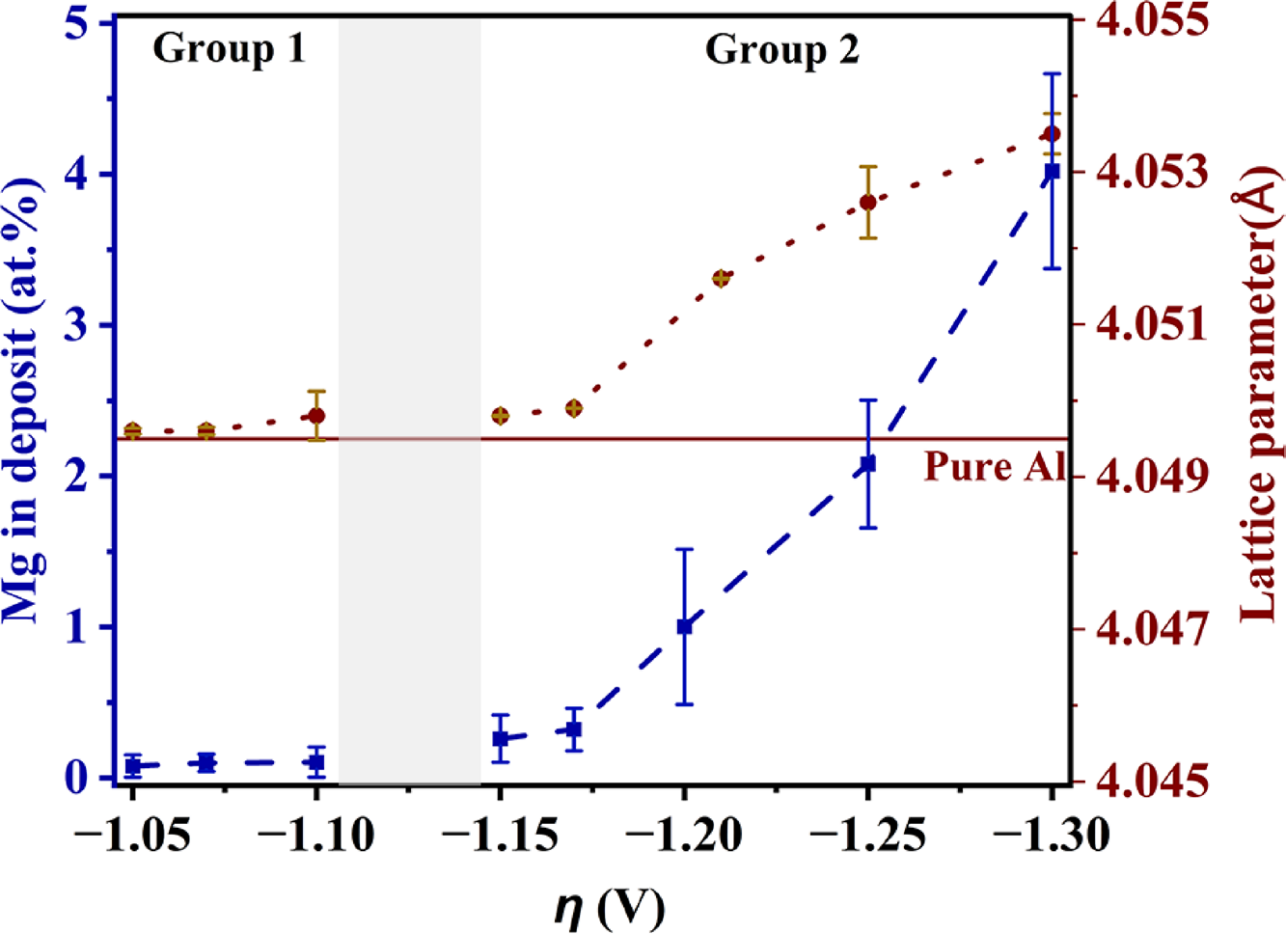
Mg content and lattice parameters of the deposits versus overpotential (*η*).

The compositional trends (Mg at.%) versus overpotential are shown in Fig. 4. From Fig. 4, the Mg content in the deposits from Group 1 is negligible and increases with overpotential in Group 2. The maximum Mg obtained in the deposits is ~ 4.02 at.%. The XRD patterns of all the deposits show the peaks corresponding to only fcc Al-phase, shown in Supplementary Fig. S3. For the sake of comparison, the XRD pattern of electrodeposited pure Al is also given in Supplementary Fig. S3. These XRD patterns do not exhibit any preferred orientation of specific crystallographic planes. From the equilibrium phase diagram of Al-Mg^33^, an intermetallic phase viz. Al_3_Mg_2_ is expected at ≤ 4 at.%. The absence of Al_3_Mg_2_ indicates the supersaturated nature of Al–Mg alloys. The lattice parameters of these pure Al and supersaturated Al–Mg alloys were estimated using Rietveld refinement as described in Supplementary Information Section S4 and shown in Supplementary Table S1 and Fig. 4. The diffraction angles of all the planes for all the deposits are furnished in Supplementary Tables S2–S10. From Fig. 4 the lattice parameter increases with Mg in the deposit. This is expected because Mg is larger than Al in size and substitutes for Al in fcc Al lattice. Interestingly, the lattice parameter change is not as drastic at higher overpotentials of Group 2 as it is at lower overpotentials within the same group (Fig. 4). This suggests a possible limit on the content of Mg that can substitute in fcc Al lattice under the present conditions. Depositions at overpotentials higher than − 1.30 V are not reported here as the deposits possess lower adherence with the substrate.

**Fig. 5:**
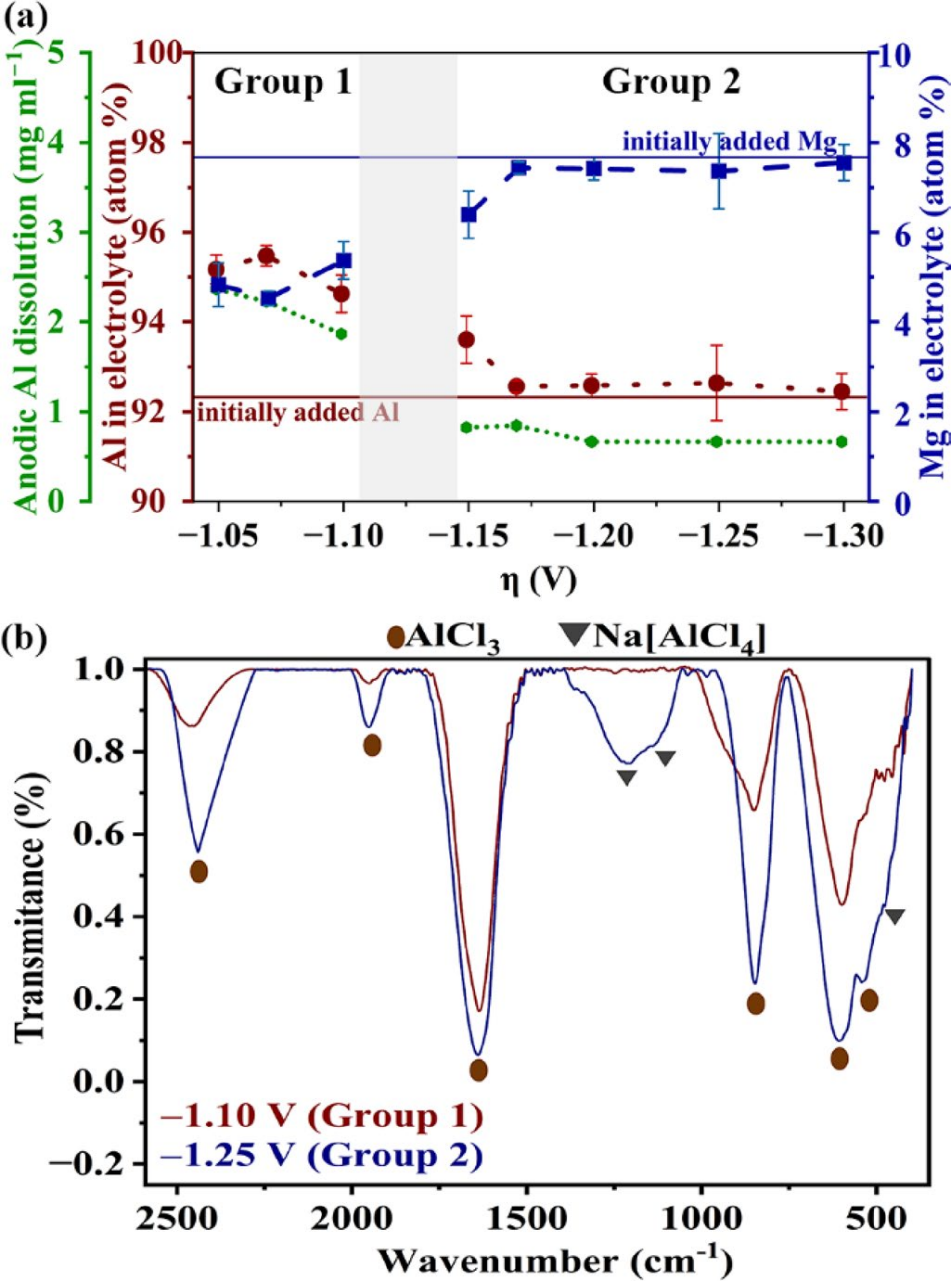
(**a**) Al and Mg in spent electrolytes and anodic dissolution of Al versus overpotential (*η*); (**b**) Representative FTIR spectra of the salts settled on anode after electrodeposition at − 1.10 V (Group 1) and − 1.25 V (Group 2).

Normally, composition of deposited alloy depends on potential (Fig. 4), temperature, electrolyte agitation, electrolyte composition, pH, etc^34^. Here, temperature was constant (180 °C) and electrolyte was not agitated. The pH is irrelevant here as the electrolytes are non-aqueous. Hence, electrolyte composition is the main factor influencing the deposit composition. The direct analysis of the spent electrolyte composition is not feasible due to the highly reactive nature of the electrolyte when in contact with atmosphere. Hence, the spent electrolytes were neutralized and their compositions were measured using Wavelength Dispersive Spectroscopy (WDS) (see Methods section). The Al at.% in the spent electrolyte was estimated as $\frac{{[Al]}_{\text{electrolyte}} \times 100}{{(\left[Al\right]+[Mg])}_{\text{electrolyte}}}$. The estimated Al and Mg contents in the electrolyte before and after deposition are plotted versus overpotential in Fig. 5a. The Al and Mg contents after deposition are mutually complementary (due to the way they were estimated as explained above). The depositions at all the overpotentials were conducted using the same initial concentrations of AlCl_3_ and MgCl_2_ in the electrolyte. Hence, the initial Al and Mg contents before deposition are the same at all the overpotentials (straight lines, Fig. 5a). Interestingly, the Al content after deposition (“Al in electrolyte (atom%)”) is higher than the initial content mainly in Group 1 depositions and is only slightly above its initial content in Group 2. In the present study anodic dissolution of Al was observed during deposition. Such anodic dissolution of Al was also reported by Huan et al^26^. The content of this dissolved Al is estimated from anodic weight loss and is also shown in Fig. 5a (“Anodic Al dissolution (mg ml^−1^)”). Hence, the Al content in the spent electrolyte (i.e. “Al in electrolyte (atom%)”) shown in Fig. 5a is a net value (= initially added Al content + anodically dissolved Al content − deposited Al content). The trends in the deposited Al, dissolved Al and Al in the spent electrolyte are also shown in Supplementary Fig. S5 in mol ml^−1^. The anodic dissolution is higher in Group 1 than that in Group 2 (Fig. 5a), where it is almost negligible. The Mg content in the spent electrolyte was also estimated by WDS and presented in Fig. 5a. The estimated Mg content in the electrolyte appears to be significantly lower than the initially added value in Group 1. This is a result of the anodic dissolution of Al in the electrolyte rather than the Mg deposition, which is negligible in Group 1 (Fig. 4). The anodic dissolution of Al slightly increases the relative percentage of Al in the spent electrolyte, which has an effect of decreasing the relative percentage of Mg. Interestingly, the Al and Mg contents in the electrolyte are almost the same as their initially added contents in the electrolyte in Group 2. This is due to the negligible anodic dissolution of Al in Group 2 and also the minimal Mg deposition (a maximum of only ~ 4.02 at.% Mg deposited at − 1.30 V, Fig. 4). Moreover, the values of deposited and dissolved Al are much lower (by an order of ~ 10^4^, when expressed as mol ml^−1^, Supplementary Fig. S5) when compared with that from the initially added Al. This implies that the bulk electrolyte composition appears nearly constant, despite getting slightly enriched in Al content. Different trends in *i*–*t* curves (Fig. 2a–c), different extents of anodic dissolution (Fig. 5a) and compositional trends (Fig. 4) in groups 1 and 2 suggest that the schemes of deposition could be different in these groups. Schemes of deposition involve phenomena occurring at both cathode (as discussed in this section) and those at anode.

### Phenomena occurring at anode

At the end of every deposition an adherent salt was observed to form over anode. Such salt formation was also observed by Holleck and Giner^35^ using 57.5AlCl_3_–12.5NaCl–20KCl electrolyte and by Wang and Hussey^36^ using 30LiAlBr_4_–50Na(AlCl_4_)–20KAlCl_4_. These studies reported that the salt passivates anode during deposition and minimizes further anodic dissolution. Here, the adhered salts on anode at the end of the depositions at representative overpotentials of − 1.10 V and − 1.25 V from both groups were analyzed by FTIR spectroscopy and the spectra are shown in Fig. 5b. These spectra indicate that the adherent salt is mainly AlCl_3_, consistent with the other reports from literature^37^. Interestingly, the FTIR spectrum of the salt from Group 2 possesses an additional peak at ~ 1220 cm^−1^ corresponding to the vibrations in Na(AlCl_4_)^38^. Na(AlCl_4_) precipitation would not have occurred during electrolyte preparation. This is because, had Na(AlCl_4_) been precipitating in the electrolyte, it would have been observed on anode after depositions within Group 1 also—which is not the case here. According to the ternary phase diagram of AlCl_3_-NaCl-KCl^39^, the expected stable phases in the vicinity of anode (containing AlCl_3_ precipitate, as mentioned above) are AlCl_3(s)_ (characterized by FTIR, Fig. 5b) and liquid at 180 °C (i.e. deposition temperature). This liquid contains Na(AlCl_4_)_(l)_, which mostly solidifies over anode upon its removal from the cell (at 180 °C) and cooling down to ambient temperature^40^. Mostly, Na^+^ approaches towards AlCl_4_^−^ formed by the reaction between AlCl_3_ forming at anode and Cl^−^ present near anode. The solidified Na(AlCl_4_) facilitates its characterization (Fig. 5b). Holleck and Giner^35^ attributed the formation of salt to the compositional stability of melt, rather than to potential, similar to the present work. This could be a reason for the formation of Na(AlCl_4_) in Group 2 and not in Group 1. If the “salt” is just a solidified electrolyte after electrodeposition, then its FTIR spectra should also show the presence of KCl, NaCl and MgCl_2_. Further, the FTIR spectra of the salts from groups 1 and 2 should be identical. Both of which are not the cases here. Hence, the salt is formed mostly during electrodeposition.

The adherent salt forming over anode can cause anodic passivation, which was investigated by recording polarization curves from OCP to positive potentials at 10 mV s^−1^ in an electrochemical cell with Cu and Al as C.E. (cathode) and W.E. (anode), respectively. The polarization curve is shown in Supplementary Fig. S6a and exhibits primary passivation (*E*_pp_ ≈ + 0.31 V vs Al). It also exhibits regions corresponding to “Decreased anodic activity” and “Pseudo passivation”. The polarization curve is also plotted in linear scale in Supplementary Fig. S6b. Supplementary Fig. S6b is slightly similar to the anodic segment of CVs shown in Fig. 1. However, a direct comparison of the anodic features from Supplementary Fig. S6b and Fig. 1 is difficult as the substrates are different. Eventually, *i*–*t* curves were recorded at representative potentials of + 0.70 and + 1.55 V vs Al|Al(III) within these regions up to 600 s and shown in Supplementary Fig. S6c. Subsequently, salt formation over Al and a deposit over Cu were observed (insets, Supplementary Fig. S6c). The FTIR spectra of these salts shown in Supplementary Fig. S6d confirm the presence of AlCl_3_ at + 0.70 V vs Al|Al(III) (region of “Decreased anodic activity”); and that of AlCl_3_ and Na(AlCl_4_) at + 1.55 V vs Al|Al(III) (region of “Pseudo passivation”). Further, the deposits over Cu at these potentials possess ~ 0 and ~ 1.47 at.% Mg, respectively. This combined analysis shows that these regions viz. “Decreased anodic activity” and “Pseudo passivation” correspond to groups 1 and 2, respectively.

Such anodic passivation can be subjective to the relative area of anode (i.e. anode/cathode area ratio), which can introduce variability in the results. Hence, potentiostatic experiments were performed at the same representative overpotential of Group 2 (− 1.25 V) with Al anodes (C.E.) of different anode areas (Supplementary Table S11). The corresponding *i*–*t* curves are shown in Supplementary Fig. S7a. From Supplementary Fig. S7a, the cathodic current densities do not change up to the anode/cathode area ratio of 19.2 times. Thus, up to this ratio the variabilities introduced by anode size are minimal in the system. However, higher cathodic current densities are obtained at further higher anode/cathode area ratio of 34.6. This suggests a lower passivation on the anode at this electrode ratio. The FTIR results on the salts forming over these anodes are shown in Supplementary Fig. S7b, and indicate the formation of AlCl_3_ and Na(AlCl_4_). A summary of the results from these potentiostatic deposition experiments are shown in Supplementary Table S11. Supplementary Fig. S7 and Table S11 show that Na(AlCl_4_) forms irrespective of the anode areas used at this representative potential. However, its quantification was not possible from FTIR. Nevertheless, the higher steady-state current densities in the *i*–*t* curves (Fig. S7a) with higher relative anode areas suggest lower passivation and possibly lower Na(AlCl_4_) formation. Supplementary Table S11 also shows that Mg deposition is lower at higher relative anode areas. Hence, at further higher anode areas Mg deposition is expected to be negligible. Depositions at further higher anode areas could not be performed due to the size limitations of the employed electrochemical cell.

### Scheme of deposition

**Fig. 6:**
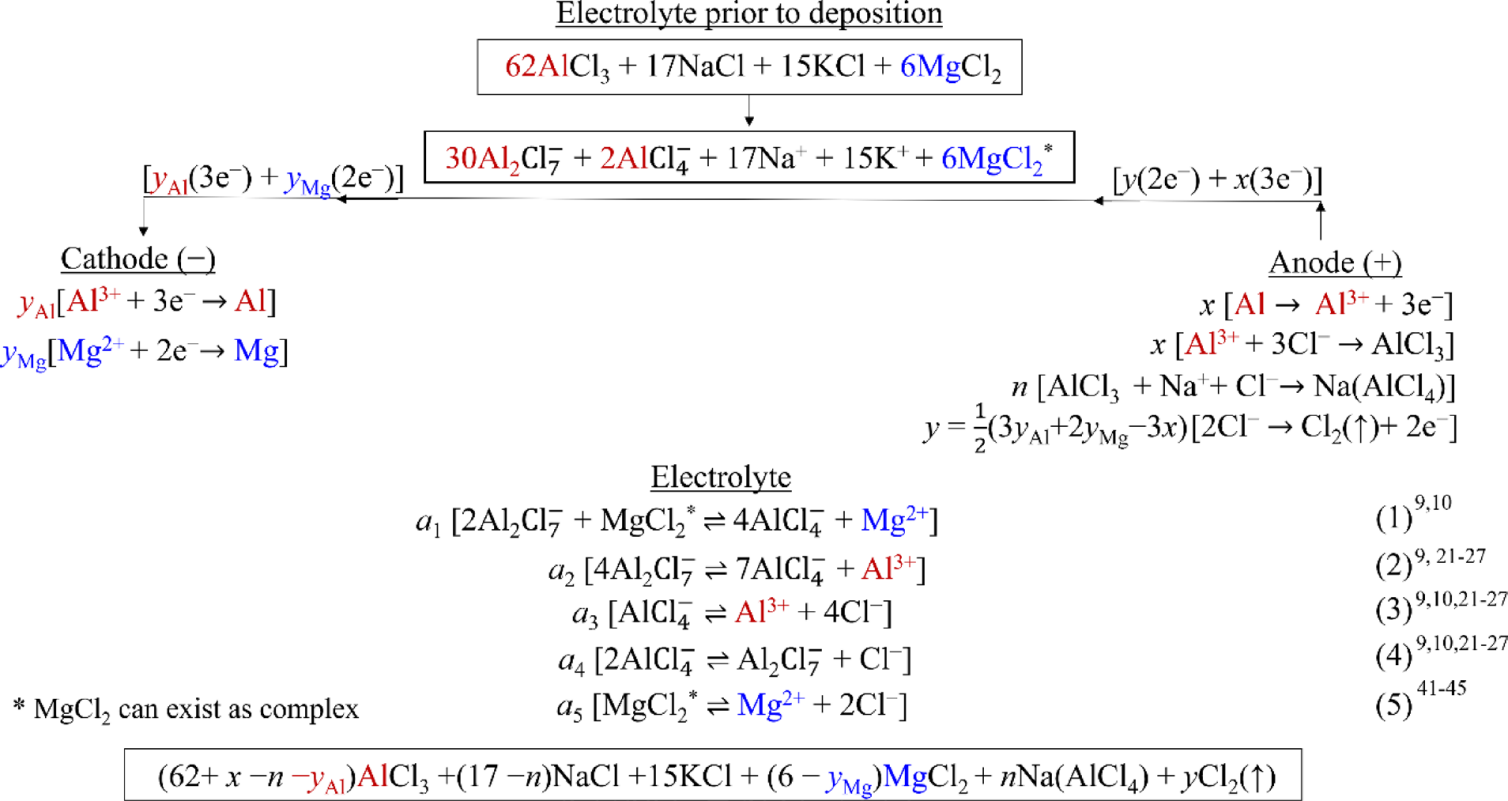
Proposed scheme for Al–Mg electrodeposition using chloride-based electrolyte employing Al as anode.

The scheme of electrodeposition should involve the changes in the electrolytes, deposits, and both electrodes. A generic scheme from the present study is proposed in Fig. 6. Various reactions (electrochemical or otherwise), possibly occurring at cathode, anode, and electrolyte (Eq. (1–5)) are shown in Fig. 6. All these reactions are well established in the literature^21–27^. Mathematical variables for cathodic reactions showing the moles of Al and Mg deposited are represented as *y*_Al_ and *y*_Mg_, respectively in Fig. 6. Similarly, variables denoting anodic dissolution (*x*), AlCl_3_ formation (*x*), Na(AlCl_4_) formation (*n*), and Cl_2_ evolution (*y* = ½(3*y*_Al_ + 2*y*_Mg_ − 3*x*)) are shown in Fig. 6. Also, the possible reactions within the electrolyte i.e. Equation (1–5) are supplied with variables *a*_j_ (j = 1–5) (Fig. 6). All these variables can take different values in groups 1 and 2.

The Cl_2_ evolution reaction given in Fig. 1 and 6 is $2{\text{Cl}}^{-}\rightleftharpoons {\text{Cl}}_{2}\left(\uparrow \right)+2{e}^{-}$. This reaction is represented by Hong-Min et al^21^ as ${4\text{AlCl}}_{4}^{-}\rightleftharpoons {{2\text{Al}}_{2}\text{Cl}}_{7}^{-}+ {\text{Cl}}_{2}\left(\uparrow \right)+2{e}^{-}$. Their reaction is a combination of $2{\text{Cl}}^{-}\rightleftharpoons {\text{Cl}}_{2}\left(\uparrow \right)+2{e}^{-}$ and Eq. (4) shown in Fig. 6. The representation by Hong-Min et al^21^ is phenomenological and is a result of ${\text{AlCl}}_{4}^{-}$ going to anode and getting oxidized and in turn causing Cl_2_ evolution. Whereas, the Cl_2_ evolution reaction given in Figs. 1 and 6 is a simplified version to render the scheme more explicable.

It is to be noted that MgCl_2_ can exist as complex(es)^11,25^. Moreover, Mg can exist as ${\text{MgCl}}_{3}^{-}$ and ${\text{MgCl}}_{4}^{2-}$ in the electrolyte^11^. However, the presence of ${\text{MgCl}}_{3}^{-}$ and ${\text{MgCl}}_{4}^{2-}$ is neglected here, due to unavailability of experimental/characterization facilities. The initial electrolyte composition translates to ${30}{\text{Al}}_{2}{\text{Cl}}_{7}^{-}$+${\text{2Al}}{\text{Cl}}_{4}^{-}$+17Na^+^ + 15K^+^ + 6MgCl_2_ (Fig. 6) considering that AlCl_3_ disproportionate to ${\text{Al}}_{2}{\text{Cl}}_{7}^{-}$ and ${\text{Al}}{\text{Cl}}_{4}^{-}$ in the presence of NaCl and KCl^21–27^. KCl improves the electrolyte conductivity by supplying Cl^−^, and decreases its viscosity by lowering its melting point.

The Al–Mg deposition scheme is proposed under the following assumptions: (i) the dissolving Al (Fig. 5a) eventually forms AlCl_3_ at anode (Fig. 5b), (ii) a part of this AlCl_3_ can react with Na^+^ and Cl^−^ to form Na(AlCl_4_) at anode (Fig. 5b), (iii) the sources of Al deposition are ${\text{Al}}_{2}{\text{Cl}}_{7}^{-}$ and Al ${\text{Cl}}_{4}^{-}$ in the electrolyte (Fig. 1, Eq. (2,3)), (iv) sources of Mg deposition are the reaction between ${\text{Al}}_{2}{\text{Cl}}_{7}^{-}$ and MgCl_2_ (Eq. (1))^9–11,25^, and the dissolution of MgCl_2_ in the electrolyte (Eq. (5)^41–45^, (v) all the net Al^3+^ and Mg^2+^ formed in electrolyte eventually deposit at cathode, (vi) all the Cl^−^ generated (Eq. (3–5)) in the electrolyte (excluding that already present as NaCl and KCl) contributes to both AlCl_3_ formation (Fig. 5b) and Cl_2_ evolution at anode (Fig. 1a)^21,24^, (vii) formation of some Mg-complexes is neglected.

The experimental results of the present study are summarized here. Al anodically dissolves (*x*) significantly in Group 1 and negligibly in Group 2 (Fig. 5a). Mg deposition (*y*_Mg_) is significant only in Group 2 (Fig. 4). Na(AlCl_4_) forms (*n*) on anode only in Group 2 (Fig. 5b). Hence, the generic scheme in Fig. 6 can be made applicable to groups 1 and 2 by adjusting the values of these variables. The schemes specific to groups 1 and 2 are discussed separately here.

In Group 1, the observed Al dissolution, no formation of Na(AlCl_4_) at anode (Fig. 5a, b), and negligible Mg deposition at cathode (Fig. 4) leads to *x* > 0, *n*≈0, and *y*_Mg_≈0, respectively. Also, no Cl_2_ evolution occurs at anode (CV in the absence of MgCl_2_, Fig. 1), leading to *y* = 0. Hence, the expression *y* = ½(3*y*_Al_ + 2*y*_Mg_ − 3*x*) reduces to *y*_Al_≈*x*. Assumption (v) with respect to Al^3+^ deposition leads to *a*_2_ + *a*_3_ = *y*_Al_ (Al^3+^ charge and mass balance). This suggests that Eq. (2–3) in Fig. 6 supports Al deposition (Fig. 4). Interestingly, assumption (v) with respect to Mg^2+^ deposition results in *a*_1_ + *a*_5_ = *y*_Mg_≈0 (as Mg deposition is negligible in Group 1). This implies that Eq. (1,5) in Fig. 6 mutually balances as Mg^2+^ produced in one equation is consumed in the other, resulting in negligible deposition of Mg (Fig. 4). Moreover, such a balance between Eq. (1) and Eq. (5) results in ${\text{2Al}}_{2}{\text{Cl}}_{7}^{-}$ + 2Cl^−^ $\rightleftharpoons$ ${\text{4Al}}{\text{Cl}}_{4}^{-}$, which is thoroughly established in the literature^24,27^. This serves as the validation of the present scheme. Similarly, assumption (vi) leads to − *3a*_2_ + *a*_3_ + *a*_4_ + *2a*_5_ = 0, demonstrating the balance among Eq. (2–5).

In Group 2, Al dissolution is negligible at anode: *x* <  < 1 (Fig. 5a). Nevertheless, according to assumption (i) the AlCl_3_ formation at anode (Fig. 5b) necessitates *x* > 0. Mg deposits by releasing Mg^2+^ from MgCl_2_ according to Eq. (1,5). Thus, a balance between these equations does not exist unlike in Group 1, and excess Cl^−^ releases (Eq. (3–5)). A part (i.e. *n*) of the released Cl^−^ goes to anode to react with *n*AlCl_3_ that is forming over anode and *n*Na^+^ to form *n*Na(AlCl_4_). This *n*Na(AlCl_4_) can passivate anode (Supplementary Fig. S6a, S6d) minimizing Al dissolution in Group 2 (Fig. 5a). Similar results were reported by Wang and Hussey^36^, which demonstrates that Al dissolution is minimal in the presence of such passivation phenomena, and vice versa. The significant formation of Na(AlCl_4_) at anode (Fig. 5b) and Mg deposition at cathode (Fig. 4) render *n* > 0 and *y*_Mg_ > 0, respectively. Interestingly, the negligible formation of net Al^3+^ at anode (*x* <  < 1), results in *y* = ½(3*y*_Al_ + 2*y*_Mg_ − 3*x*) > 0, supporting Cl_2_ evolution (CV in the presence of MgCl_2_, Fig. 1). In other words, Cl_2_ evolution at anode almost entirely serves as the source of electrons for Al–Mg deposition in Group 2. Similar to Group 1, assumption (v) pertaining to Al^3+^ deposition (Fig. 4) leads to *a*_2_ + *a*_3_ = *y*_Al_. This assumption, with respect to Mg^2+^ deposition, leads to *a*_1_ + *a*_5_ = *y*_Mg_. Here, assumption (vi) results in 4*a*_3_ + *a*_4_ + 2*a*_5_ = *n* + *3y*_Al_ + *2y*_Mg_ for balancing Eq. (3–5), the equations for Na(AlCl_4_) formation and those for Al, Mg depositions.

Upon various consumptions and productions of (electro)chemical species during Al–Mg deposition through the generic scheme (Fig. 6), the remaining electrolyte would possess the composition: (62 + *x − n − y*_Al_)AlCl_3_ + (17 − *n*)NaCl + 15KCl + (6 − *y*_Mg_)MgCl_2_ + *n*Na(AlCl_4_) + *y*Cl_2_(↑).

### Corollaries from the scheme of deposition

Various important corollaries can be derived from the generic scheme of Al–Mg deposition presented above (Fig. 6). For comparing the same variables of Groups 1 and 2, they are abbreviated as *G*_1_ and *G*_2_, respectively.

#### Corollary 1: Al deposits preferentially in Group 1

The total charge transferred to cathode during deposition in groups 1 and 2 may not be the same. This leads to a possible consequence of the total concentration of Cl^−^ going to anode being the same in these groups. In Group 1 this is $3{x|}_{{G}_{1}}(=3{{y}_{\text{Al}}|}_{{G}_{1}})$ (since ${n|}_{{G}_{1}},{{2y}_{\text{Mg}}|}_{{G}_{1}}$≈0, Figs. 5b, 4 and 6); and in Group 2 this is ${{3y}_{\text{Al}}|}_{{G}_{2}}+ {{2y}_{\text{Mg}}|}_{{G}_{2}}+{n|}_{{G}_{2}}$. Equating these two quantities and rearranging yields: $3{{y}_{\text{Al}}|}_{{G}_{1}}$− ${\text{3}}{{y}_{\text{Al}}|}_{{G}_{2}}$ =${\text{2}}{{y}_{Mg}|}_{{G}_{2}}+ {n|}_{{G}_{2}}>0$. Thus, Al deposits more in Group 1 than in 2. 

The possibility of the total charge transfer being the same in groups 1 and 2 can be expressed mathematically as ${{3y}_{\text{Al}}|}_{{G}_{1}}+ {{2y}_{\text{Mg}}|}_{{G}_{1}}={{3y}_{\text{Al}}|}_{{G}_{2}}+ {{2y}_{\text{Mg}}|}_{{G}_{2}}$. Noting that ${{2y}_{\text{Mg}}|}_{{G}_{1}}\approx 0$ (Fig. 4), this equation yields: $3{{y}_{\text{Al}}|}_{{G}_{1}}$− ${\text{3}}{{y}_{\text{Al}}|}_{{G}_{2}}$ = ${\text{2}}{{y}_{\text{Mg}}|}_{{G}_{2}}>0$. Interestingly, here also Al deposits more in Group 1 than in 2.

Thus, universally Al deposits preferentially in Group 1. This is seen from Fig. 4, which shows that mainly Al deposits in Group 1, unlike Al and Mg together depositing in Group 2 (Fig. 4).

#### Corollary 2: Mg deposition is a prerequisite for Na(AlCl_*4*_) formation at anode

This corollary can be arrived at by assuming that Mg does not deposit in Group 2. By continuing from the above corollary: ${\text{2}}{{y}_{\text{Mg}}|}_{{G}_{2}}= (3{{y}_{\text{Al}}|}_{{G}_{1}}$− ${\text{3}}{{y}_{\text{Al}}|}_{{G}_{2}}$) − ${n|}_{{G}_{2}}>0$. Here, ${\text{2}}{{y}_{\text{Mg}}|}_{{G}_{2}}=0$ as per the present assumption. It means that only Al deposits in Group 2, implying that $3{{y}_{\text{Al}}|}_{{G}_{2}}$= ${{3y}_{\text{Al}}|}_{{G}_{1}}$ (since, only Al deposits in Group 1). This necessitates ${n|}_{{G}_{2}}=0$. In other words, Na(AlCl_4_) does not form at anode in Group 2, which is absurd (Fig. 5b). Thus, Mg deposition is a prerequisite for Na(AlCl_4_) formation at anode. This also substantiates the justification for the formation of Na(AlCl_4_) discussed in the Sect. “Scheme of deposition”.

Moreover, the deposition experiments at − 1.25 V (Group 2) with the higher relative area of anode showed that Mg in these deposits decreases as the area ratio increases (Supplementary Table S11). According to this corollary, deposition at further higher anode relative areas may not result in Mg deposition hence ceasing the formation of Na(AlCl_4_).

#### Corollary 3: formation of Na(AlCl_4_) curtails amount of Mg deposited from increasing beyond its maximum in Group 2

Invoking the same equation from Corollary 1, ${{2y}_{\text{Mg}}|}_{{G}_{2}}= (3{{y}_{\text{Al}}|}_{{G}_{1}}$− ${{3y}_{\text{Al}}|}_{{G}_{2}}$) − ${n|}_{{G}_{2}}>0$, the maximum amount of Mg that can be deposited is 2 ${{y}_{\text{Mg}}|}_{{G}_{2},\text{max}}=\left(3{{y}_{\text{Al}}|}_{{G}_{1}}- 3{{y}_{\text{Al}}|}_{{G}_{2},\text{min}}\right),$ assuming ${n|}_{{G}_{2}}=0$. However, from Corollary 2, Mg deposition leads to Na(AlCl_4_) formation, implying that ${n|}_{{G}_{2}}>0$. Hence the actual amount of Mg deposited (${{2y}_{\text{Mg}}|}_{{G}_{2}}$) is always less than its theoretical maximum (${{2y}_{\text{Mg}}|}_{{G}_{2},\text{max}}$) by ${n|}_{{G}_{2}}$. In other words, the formation of Na(AlCl_4_) curtails the amount of Mg deposited from increasing beyond its maximum in Group 2.

#### Corollary 4: a minimum threshold value exists for the ratio of the amounts of Al and Mg deposited in Group 2

From Corollary 1, $3{{y}_{\text{Al}}|}_{{G}_{1}}$> ${{3y}_{\text{Al}}|}_{{G}_{2}}$, implying that $2{{y}_{\text{Mg}}|}_{{G}_{1}}$< ${{2y}_{\text{Mg}}|}_{{G}_{2}}$. This means that $\frac{3{{y}_{\text{Al}}|}_{{G}_{1}}}{2{{y}_{\text{Mg}}|}_{{G}_{1}}} > \frac{3{{y}_{\text{Al}}|}_{{G}_{2}}}{2{{y}_{\text{Mg}}|}_{{G}_{2}}}$ . Alternatively, the ratio of the amounts of Al and Mg deposited in Group 1 is higher than that in Group 2. Further, considering Corollary 3, the ratio $\frac{3{{y}_{\text{Al}}|}_{{G}_{2}}}{2{{y}_{\text{Mg}}|}_{{G}_{2}}}$ is always more than $\frac{3{{y}_{\text{Al}}|}_{{G}_{2}}}{2{{y}_{\text{Mg}}|}_{{G}_{2},\text{max}}}$. This indicates that a minimum threshold value exists for the $\frac{\text{Al}}{\text{Mg}}$ ratio in the deposits in Group 2. It is difficult to establish a similar argument for the ratio in the electrolytes as the proportions of the reactions (i.e. *a*_j_ = 1–5) within the electrolyte are not known.

The proposed scheme of deposition and the corollaries can answer the questions: (i) Can a desired content of Mg be obtained in the deposits? Alternatively, can Al–Mg alloys be deposited with any composition? A desired content of Mg up to a maximum of 4.02 atom% can be deposited from the present electrolyte system. This is because Al deposits preferentially (Corollary 1). Moreover, further deposition of Mg is curtailed by the formation of Na(AlCl_4_) over anode (Corollaries 2 and 3). (ii) How can the deposit composition be controlled? From the present study, the deposit composition can be controlled by changing the overpotential and possibly by changing the counter electrode to any metal that does not chemically react with the electrolyte, such as graphite, Pt etc^22,46^.

## Methods

The electrolyte for electrodeposition of Al–Mg was prepared using AlCl_3_ (Reagent Plus®, 99%, SIGMA), NaCl (analysis grade, EMPARTA), KCl (analysis grade, EMPARTA), and MgCl_2_ (anhydrous, ≥ 98%, SIGMA). The chemicals were handled in argon (Ar) atmosphere employing MBRAUN EASYlab^pro^*i* 4-port glovebox maintaining oxygen (O_2_) and moisture (H_2_O) levels of ˂ 0.1 ppm. The electrolyte was prepared afresh prior to every experiment by melting the salts together in the given composition at 180 °C in a 10 ml beaker. The electrolyte composition for Al–Mg alloy deposition was 62 mol% AlCl_3_ + 17 mol% NaCl + 15 mol% KCl + 6 mol% MgCl_2_. The composition of the electrolyte for pure Al deposition was 61 mol% AlCl_3_ + 22 mol% NaCl + 17 mol% KCl, with the respective chemicals obtained from the above mentioned sources with respective grades. According to the ternary phase diagram of AlCl_3_–NaCl–KCl^39^ the solubilities of NaCl and KCl at 180 °C are approximately ~ 24 mol% each. Hence the added NaCl and KCl are completely soluble in AlCl_3_, maintaining a homogenous liquid phase. The electrolyte was further purified while in the molten state (i.e. at 180 °C) by dipping an Al rod (99.99% pure, PureSynth) for 24 h. At the end of this purification process, the impurities were collected by the Al rod. Eventually, the Al rod with the collected impurities was discarded. The purified electrolyte was used for electrochemical experiments. For all the electrochemical experiments, a CHI660E electrochemical workstation was employed. For deposition experiments, aluminum (Al) rod and strips were used as counter electrode (C.E.). An Al strip (99.99% pure) was used as the reference electrode (R.E.), throughout the study.

A commercial copper (Cu) rectangular strip (~ 0.35 cm^2^_,_ both sides included) was used as the working electrode (W.E.). All the experiments were conducted at 180 °C. Before every experiment, the Cu electrode was manually polished using sand papers (120C, 200C, 400C, 800C, 1000C, 2000C) followed by diamond paste (6–12 µm, 3–5 µm), alumina powder (~ 1–1.5 µm) and again diamond paste (0.5–1 µm). Similarly, the Al electrodes were prepared afresh by polishing using sandpapers (80C, 120C, 200C, 800C, 1000C). Prior to every experiment open circuit potential (OCP) was measured until it was stable. OCP measurements were done for a period of 1200 to 1800s for obtaining a stable value. In the case OCP was not stable after this period the electrolyte was discarded and the electrical wirings and circuits within the glove box were checked, and fresh electrolytes were prepared to conduct the experiments, starting from OCP measurement again. The stable OCP value in the absence of MgCl_2_ was around + 0.57 V vs Al|Al(III); and those in the presence of MgCl_2_ were between + 0.48 and + 0.49 V vs Al|Al(III).

The possible presence of all the peaks due to the electrochemical reactions in depositing Al or Al–Mg alloys was checked by cyclic voltammetry (CV). For recording CVs, the applied potential was swept from OCP to a switching potential of − 2.00 V vs Al|Al(III) and further to + 2.50 V vs Al|Al(III) on the positive side at a scan rate of 10 mVs^−1^. Al rod was employed as the counter electrode for CVs.

The potentiostatic electrodepositions were conducted for 600 s by using applied potentials between − 0.55 and − 0.85 V vs Al|Al(III) in separate experiments. For the present study overpotentials were estimated as the difference between the applied potentials and the OCPs (measured as mentioned above prior to deposition experiments). Hence, the applied potentials for Al–Mg deposition correspond to overpotentials between − 1.05 and − 1.30 V. Pure Al was deposited from the electrolyte without MgCl_2_ at an overpotential corresponding to − 1.07 V. Eventually, the working electrodes containing the Al–Mg deposits were cleaned by removing the adherent solidified molten salt through ultrasonicating in distilled water for 15 min. The cleaned deposits were sent for morphological and compositional analyses.

After every electrodeposition, a colorless salt formation was observed on the anode when the system was cooled to room temperature. The cooled-down anode was seal-packed in the glovebox and brought outside for its characterization. It is possible that this salt can cause anodic passivation. This anodic passivation behavior on the Al electrode was investigated by initially, reversing the above-mentioned electrochemical cell (i.e. Al as W.E. and Cu as C.E.) and recording the polarization curve from OCP to + 2.50 V vs Al|Al(III) at 10 mV s^−1^. Eventually, potentiostatic experiments were conducted at representative potentials belonging to certain regions from the polarization curve.

The spent electrolytes were collected in alumina crucibles inside the glove box and brought out of the glove box after they solidified for compositional analysis. Eventually, they were neutralized by adding a few drops of distilled water in a fume hood. The electrolytes were left undisturbed until they stopped fuming and turned transparent. The transparent electrolytes were then dried in a hot air oven at 120 °C for ~ 48 h and taken for compositional analysis.

All characterizations were done at ambient temperature. For morphological analysis, a JEOL FEG-SEM (JSM-7600F) Scanning Electron Microscope (SEM) was used. Wavelength Dispersive Spectrometer (WDS) detector connected to SEM was employed for compositional analysis of deposits and spent electrolytes (in neutralized condition) to avoid any possible absorption/fluorescence effects between the signals from Al and Mg. The deposited Al–Mg alloys were analyzed in as-deposited conditions. The particle sizes of the deposits were measured using ImageJ 1.54 g software. For every particle, the diameters were measured in two mutually perpendicular directions and averaged. For deposit at every overpotential the particle sizes were estimated for at least 200 of them from multiple SEM images. The arithmetic averages of these sizes and their standard deviations were used for analysis.

The phase analysis was done by employing a Rigaku X-ray diffractometer (SmartLab 9 kW, Cu *K*_*α*_, λ = 1.5406 Å, 160 mA, 45 kV). The baseline correction of the obtained results was done, and the phases were indexed using ICSD (code: 44,321), corresponding to pure Al. Rietveld refinement of the profiles was done using the software FullProf (version: 7.20) for phase identification and lattice parameter estimation. A detailed method of Rietveld refinement is given in Supplementary Information Section S4. The functional groups in the salts settled on the counter electrode were identified by employing a 3000 Hyperion Microscope with Vertex 80 Fourier-transform Infrared (FTIR) spectroscope, Bruker, Germany. The FTIR data was indexed using File card number 744-70-0, SpectraBase® (John Wiley & Sons, 1981–2024).

## Electronic supplementary material

Below is the link to the electronic supplementary material.

Supplementary Material 1

## Data Availability

The authors declare that all data supporting the findings of this study are available within the paper and its supplementary information files. Additional data related to this study are available from the corresponding author on reasonable request.
