# Supplementary material for: Electrodeposition of Al–Mg alloys from chloride-based molten salts
Source: Sci Rep. 2025 May 30;15:19092. doi: 10.1038/s41598-025-04094-1 (PMC12125349; doi:10.1038/s41598-025-04094-1)
Supplement: Supplementary file 1 — Supplementary Material 1 [file 41598_2025_4094_MOESM1_ESM.pdf]

Supplementary Information

Electrodeposition of Al-Mg alloys from chloride-based molten salts

Sreesvarna Bhaskaramohan[1], Manepalli J. N. V. Prasad[2], G. V. Dattu Jonnalagadda[3], Sankara Sarma V. Tatiparti[1,4,*]

[1]Department of Energy Science and Engineering, Indian Institute of Technology Bombay, Mumbai, 400076, India

[2]Department of Metallurgical Engineering and Materials Science, Indian Institute of Technology Bombay, Mumbai, 400076, India

[3]John F Welch Technology Centre, General Electric India Industrial Pvt. Ltd., Bangalore, 560066, India

[4]Materials Science and Engineering Department, University of Florida, Gainesville, FL 32611, USA

[*]sankara@iitb.ac.in

S1. Linear Sweep Voltammetry

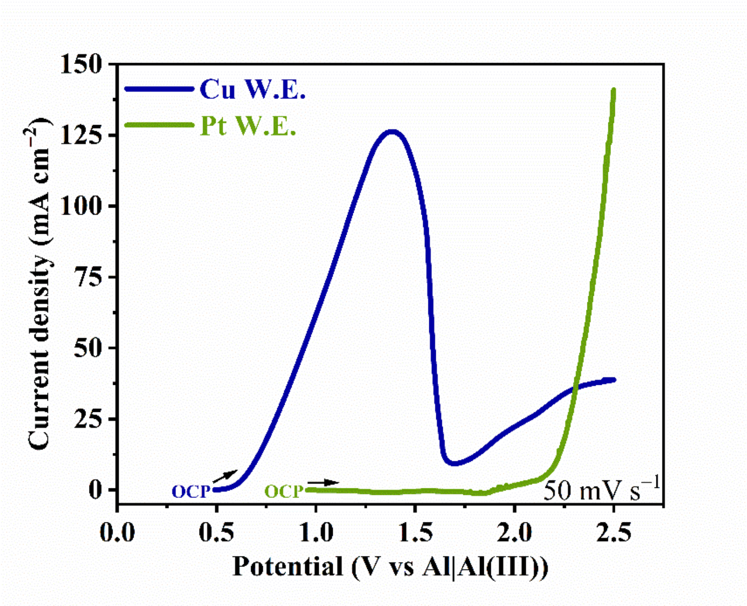

Figure S1. Linear Sweep Voltammetry on Cu and Pt electrode using $62AlCl_3 + 17NaCl + 15KCl + 6MgCl_2$ electrolyte.

S2. Current density- time (*i-t*) curves at overpotential −1.12 V

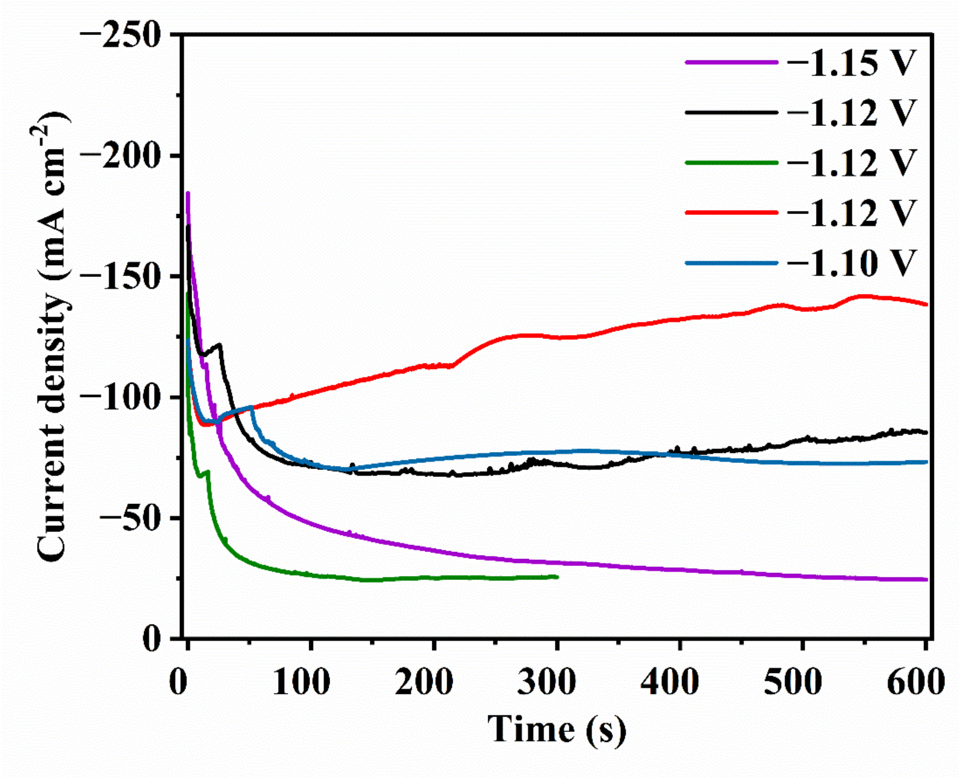

Figure S2. *i-t* curves at overpotentials −1.10 V, −1.12 V and −1.15 V.

S3. X-Ray Diffraction (XRD)

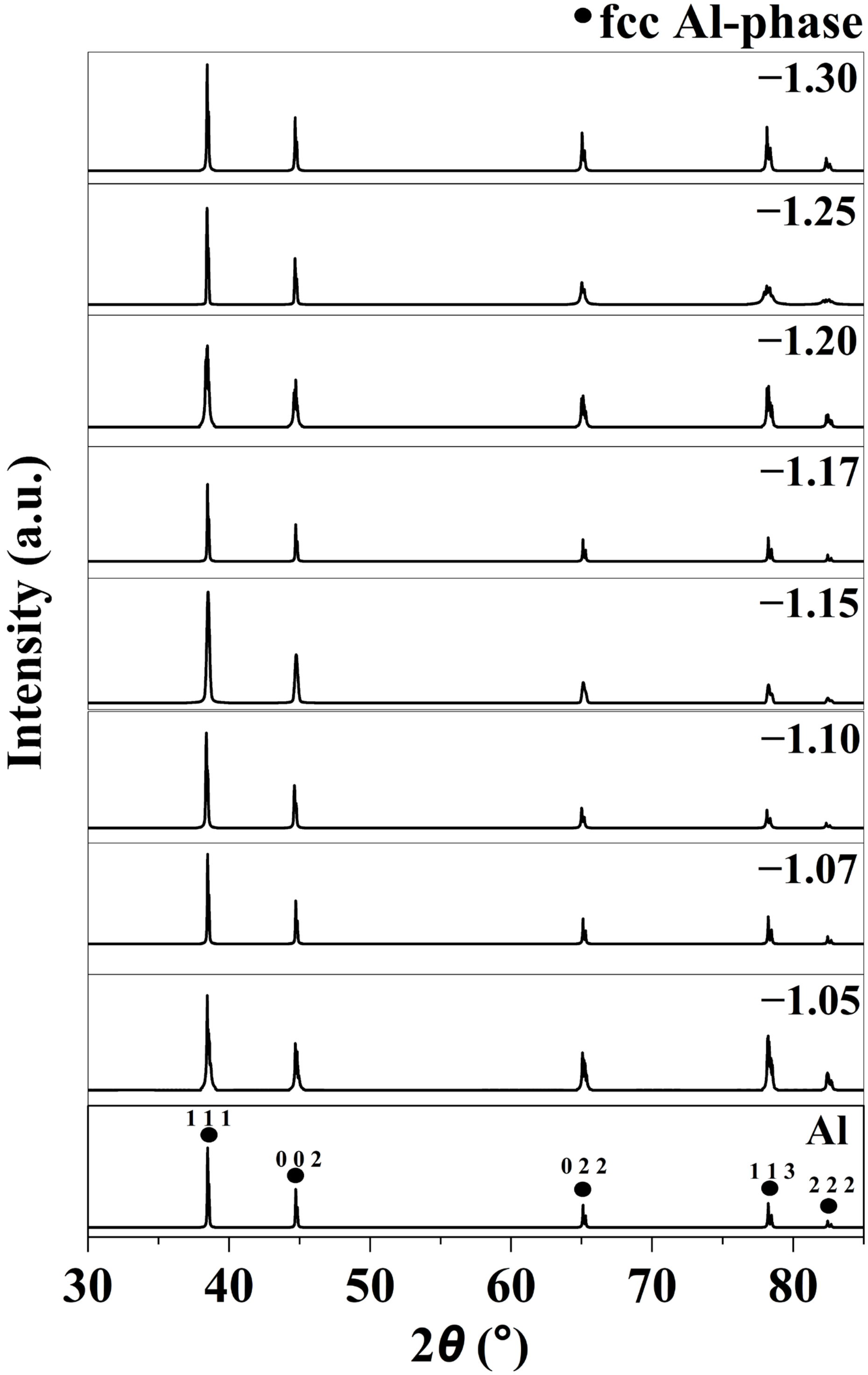

Figure S3. XRD patterns of electrodeposited pure Al and Al-Mg alloys at different overpotentials.

S4. Rietveld refinement

The XRD results obtained were used to estimate lattice parameters of the Face Centred Cubic (fcc) Al phase employing "Rietveld refinement"[1]. Prior knowledge of the crystallographic information such as space groups of phases likely to be present in the samples were used for the refinement. In the present work, fcc Al phase is present as seen from the Supplementary Fig. S3. The software, FullProf suite (version: 7.20) was employed for Rietveld refinement as it is widely used[2]. Prior to refinement, the backgrounds of the raw XRD patterns were corrected using winPLOTR program[3]. Instrumental factors and/or presence of any non-stoichiometric compound (such as amorphous Al_2O_3) may lead to this background. The space group, peak positions and lattice parameters of pure Al (standards) are obtained from the standard ICSD reference data for Al [ICSD code: 44321].

The peaks of XRD pattern generally follow Pseudo-Voigt function ($pV(x)$)[4]. This mathematical function is a linear combination of two functions namely the Gaussian ($G(x)$, Eq. (S1)) and the Lorentzian ($L(x)$, Eq. (S5)). All the parameters used in the Gaussian and Lorentzian functions are given in Eqs. (S2-S4, S6, S7). The weighed fraction of η is used for combining the Gaussian function and Lorentzian function to obtain the Pseudo-Voigt function given in Eq. (S8).

$$G(x)|_{x=-1\ to\ 1} = a_g\ exp\left(-b_g x^2\right) \tag{S1}$$

where,

$$a_G = \frac{2}{FWHM}\sqrt{\frac{ln\ 2}{FWHM^2}} \tag{S2}$$

$$b_G = \frac{4\ ln2}{FWHM^2} \tag{S3}$$

FWHM: Full width half maximum

$$FWHM^2 = \left(U + D_{ST}{}^2\right)(\tan\theta)^2 + V\tan\theta + W + \frac{I_G}{(\cos\theta)^2} \quad \text{(S4)}$$

where, U, V, W and I_G are FWHM parameters

Lorentzian function $\left(L(x)\right)$:

$$L(x)|_{x=-1\ to\ 1} = \frac{a_L}{1+b_L x^2} \quad \text{(S5)}$$

where,

$$a_L = \frac{2}{\pi FWHM} \quad \text{(S6)}$$

$$b_L = \frac{4}{FWHM^2} \quad \text{(S7)}$$

Pseudo-Voigt function $\left(pV(x)\right)$:

$$pV(x) = \eta\, L(x) + (1-\eta)G(x) \quad \text{(S8)}$$

where,

$$\eta = \eta_0 + X2\theta \quad \text{(S9)}$$

where, η_0 and X are shape parameters.

The values U, V, W, η_0 and X was varied until a minimum χ^2 fit was achieved for the refined data. The lattice parameters and minimum χ^2 achieved for every overpotential is given in Table S1. The patterns corresponding to the fitted, experimental and the residual errors obtained are shown in Fig. S4.

Table S1. Lattice parameters and minimum χ^2 achieved for every overpotential

**Overpotential, η (V)**	**Lattice parameter, *a* (Å)**	**Standard deviation of *a***	χ^2
Pure Al	4.0495	0.000954	4.315
−1.05	4.0496	0.000004	1.813
−1.07	4.0496	0.000005	1.286
−1.10	4.0498	0.000321	5.66
−1.15	4.0498	0.000004	4.522
−1.17	4.0499	0.000002	1.090
−1.20	4.0516	0.000002	1.803
−1.25	4.0526	0.000467	1.813
−1.30	4.0535	0.000264	3.002

S4.1. Structure factors

The structure factors (*F*) of the planes were estimated using the intensity (*I*) from the XRD peaks obtained after refinement. The relation between *F* and *I* is given in Eq. (S10)[5].

$$I \propto |F|^2 \tag{S10}$$

The *F* is Fourier transform possessing a unique value at each (*h k l*) reflection. This follows the relation shown in Eq. (S11)[5].

$$F(hkl) = \sum_h \sum_k \sum_l f_{Al}.exp(2\pi i \alpha_{hkl}) \tag{S11}$$

where, f_{Al} is the atomic scattering factor of Al atom and α_{hkl} is given in Eq. (S12).

$$\alpha_{hkl} = h.u + k.v + l.w \tag{S12}$$

where, $u = \frac{x}{a}; v = \frac{y}{b}; w = \frac{z}{c}$

Here, the position of Al atom is given by (x, y, z). The lattice parameters of Al unit cell are given by a, b and c (all these parameters have the same value as it is an fcc system).

The estimated d_{hkl} values and F for all deposits obtained at different overpotentials are shown in Tables (S2-S10).

Table S2. Structure factors for deposited pure Al

**2*Θ***	***h k l***	d_{hkl}	$\|F\|^2$
38.472	1 1 1	2.3380	1342.9181
44.721	0 0 2	2.0248	1215.2780
65.096	0 2 2	1.4317	905.6771
78.229	1 1 3	1.2210	754.0677
82.436	2 2 2	1.1690	711.5412

Table S3. Structure factors for deposited at −1.05 V

**2*Θ***	***h k l***	d_{hkl}	$\|F\|^2$
38.471	1 1 1	2.3381	1342.9528
44.719	0 0 2	2.0248	1215.3114
65.094	0 2 2	1.4318	905.7131
78.225	1 1 3	1.2210	754.1051
82.432	2 2 2	1.1690	711.5789

Table S4. Structure factors for deposited at −1.07 V

2θ	$h\ k\ l$	d_{hkl}	$\|F\|^2$
38.472	1 1 1	2.3380	1342.9208
44.720	0 0 2	2.0248	1215.2805
65.096	0 2 2	1.4317	905.6800
78.229	1 1 3	1.2210	754.0706
82.435	2 2 2	1.1690	711.5441

Table S5. Structure factors for deposited at −1.10 V

2θ	$h\ k\ l$	d_{hkl}	$\|F\|^2$
38.469	1 1 1	2.3382	1342.9991
44.716	0 0 2	2.0249	1215.3568
65.090	0 2 2	1.4319	905.7606
78.220	1 1 3	1.2211	754.1550
82.427	2 2 2	1.1691	711.6294

Table S6. Structure factors for deposited at −1.15 V

2θ	$h\ k\ l$	d_{hkl}	$\|F\|^2$
38.469	1 1 1	2.3382	1342.9938
44.717	0 0 2	2.0249	1215.3518
65.090	0 2 2	1.4318	905.7552
78.221	1 1 3	1.2211	754.1494
82.427	2 2 2	1.1691	711.6236

Table S7. Structure factors for deposited at −1.17 V

2Θ	*h k l*	d_{hkl}	$\lvert F\rvert^2$
38.469	1 1 1	2.3382	1342.9829
44.717	0 0 2	2.0249	1215.3412
65.091	0 2 2	1.4318	905.7440
78.222	1 1 3	1.2211	754.1379
82.428	2 2 2	1.1691	711.6119

Table S8. Structure factors for deposited at −1.20 V

2Θ	*h k l*	d_{hkl}	$\lvert F\rvert^2$
38.453	1 1 1	2.3391	1343.3510
44.698	0 0 2	2.0257	1215.6993
65.061	0 2 2	1.4324	906.1234
78.184	1 1 3	1.2216	754.5348
82.387	2 2 2	1.1695	712.0123

Table S9. Structure factors for deposited at −1.25 V

2Θ	*h k l*	d_{hkl}	$\lvert F\rvert^2$
38.442	1 1 1	2.3398	1343.6012
44.685	0 0 2	2.0263	1215.9435
65.041	0 2 2	1.4328	906.3813
78.158	1 1 3	1.2219	754.8050
82.360	2 2 2	1.1699	711.2846

Table S10. Structure factors for deposited at −1.30 V

**2*Θ***	***h k l***	d_{hkl}	$\lvert F \rvert^2$
38.434	1 1 1	2.3403	1343.7908
44.675	0 0 2	2.0267	1216.1285
65.026	0 2 2	1.4331	906.5770
78.139	1 1 3	1.2222	755.0096
82.339	2 2 2	1.1701	712.4914

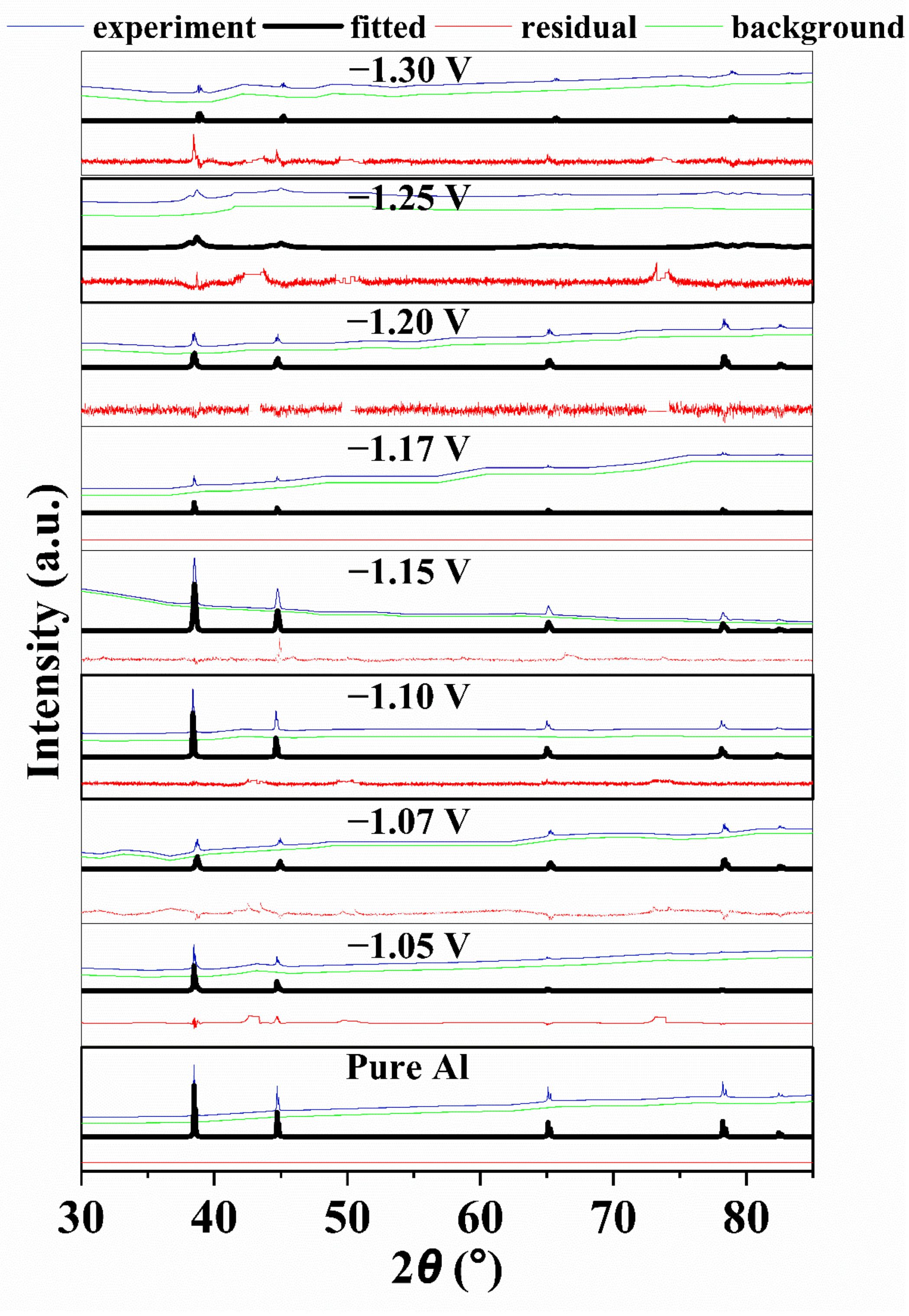

Figure S4. Fitted XRD patterns after Rietveld refinement of deposits at different overpotentials.

S5. Trends in deposited Al, Al anodic dissolution and Al in spent electrolyte

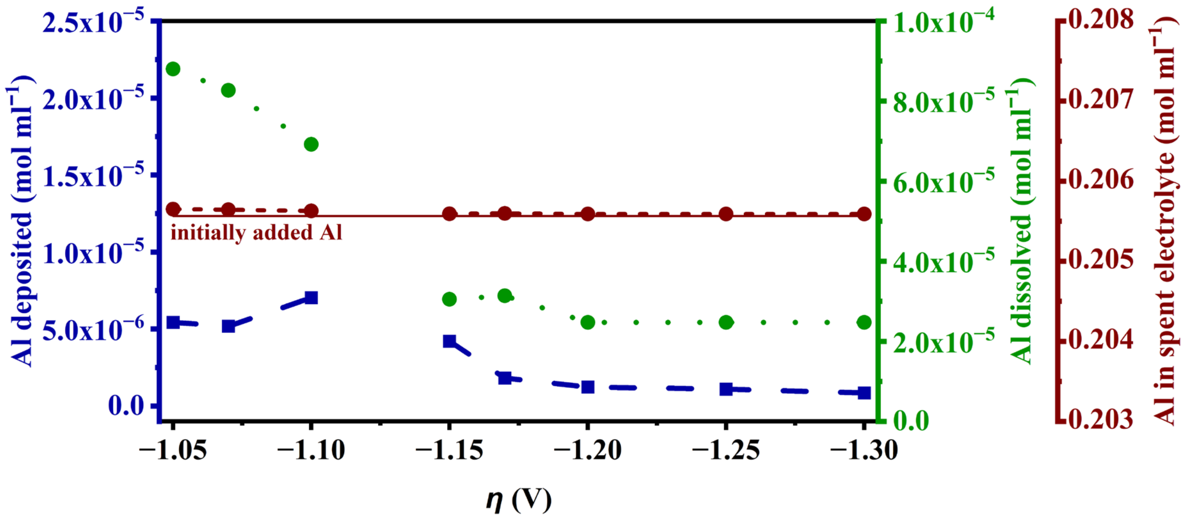

Figure S5. Trends in deposited Al, Al anodic dissolution and Al in spent electrolyte.

S6. Phenomena occurring at anode

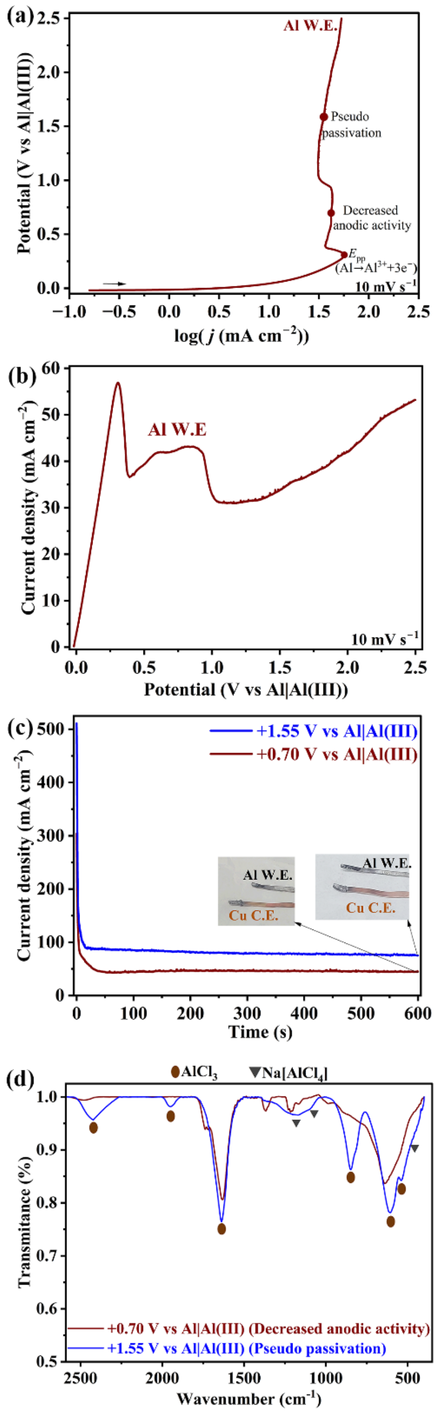

Figure S6. (**a**) Polarization curves of Al W.E.; (**b**) Linear Sweep Voltammetry on Al W.E; (**c**) Current density-time curves at applied potentials +0.70 and +1.55 V vs Al|Al(III) chosen from (a); (**d**) FTIR spectra of the salts on anode at the end of experiments in (c).

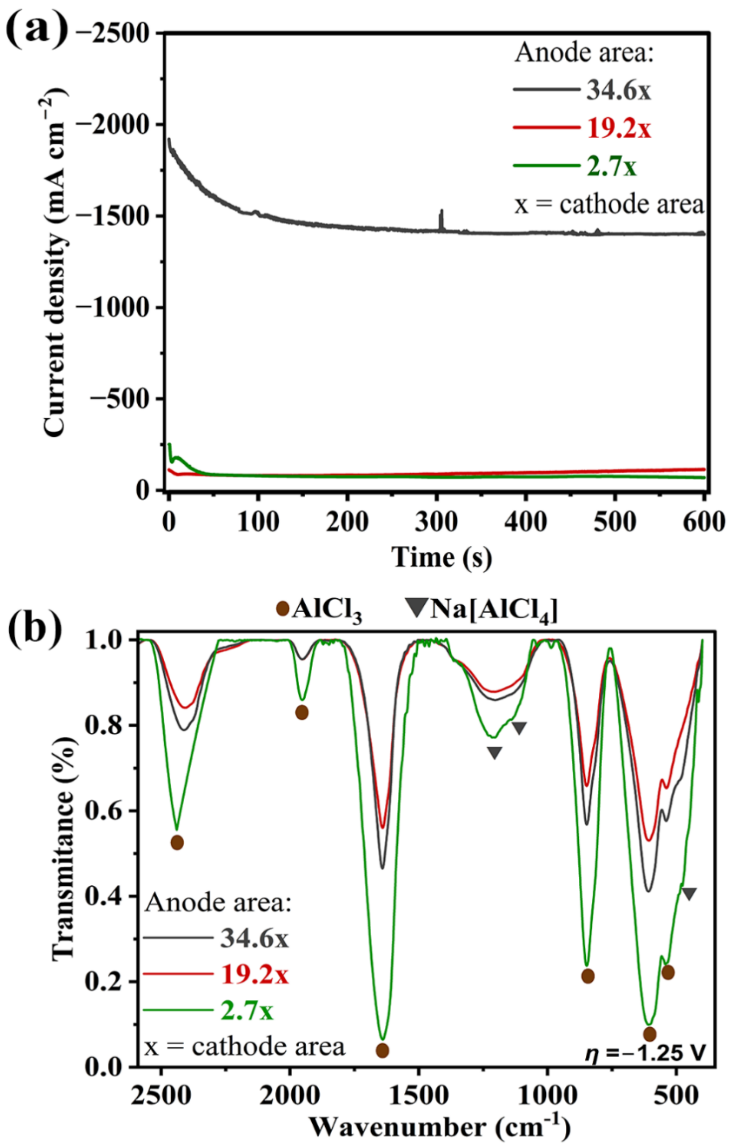

Figure S7. (**a**) *i-t* curves with different counter electrode areas at −1.25 V overpotential, (**b**) FTIR results on the salts forming over the anodes with different areas.

Table S11. Summary of results from potentiostatic depositions with various anode/cathode areas

(Al anode) / (Cu cathode) area ratio	Al anode area relative to the smallest anode area used	Al dissolution (mg ml^{-1})	Na($AlCl_4$) formed?	Mg in deposit (at.%)
**2.7**	1 (≈2.7/2.7)	0.7	Yes	2.08
**19.2**	7.1 (≈19.2/2.7)	2.9	Yes	1.13
**34.6**	12.8 (≈34.6/2.7)	5.3	Yes	0.89

References

1. McCusker, L. B., Von Dreele, R. B., Cox, D. E., Louër, D. & Scardi, P. Rietveld refinement guidelines. *J. Appl. Crystallogr.* **32**, 36–50 (1999).

2. Rodrıguez-Carvajal, J. FULLPROF, a program for Rietveld refinement and pattern matching analyses. *Satellite Meeting on Powder Diffraction of the XVth Congress of the International Union of Crystallography* **127**, (1990).

3. Roisnel, T. & Rodríquez-Carvajal, J. WinPLOTR: a windows tool for powder diffraction pattern analysis. in *Materials Science Forum* **378**, 118–123 (Transtec Publications, 2001).

4. Young, R. A. & Wiles, D. B. Profile shape functions in Rietveld refinements. *J. Appl. Crystallogr.* **15**, 430–438 (1982).

5. Cullity, B. D. *Elements of X-ray Diffraction.* (Addison-Wesley Publishing, 1956).
